# Prostatitis, Sexually Transmitted Diseases, and Prostate Cancer: The California Men's Health Study

**DOI:** 10.1371/journal.pone.0008736

**Published:** 2010-01-15

**Authors:** Iona Cheng, John S. Witte, Steven J. Jacobsen, Reina Haque, Virginia P. Quinn, Charles P. Quesenberry, Bette J. Caan, Stephen K. Van Den Eeden

**Affiliations:** 1 Epidemiology Program, Cancer Research Center of Hawai'i, University of Hawai'i, Honolulu, Hawai'i, United States of America; 2 Department of Epidemiology and Biostatistics and Institute of Human Genetics, University of California San Francisco, San Francisco, California, United States of America; 3 Department of Research and Evaluation, Kaiser Permanente Southern California, Pasadena, California, United States of America; 4 Division of Research, Kaiser Permanente Northern California, Oakland, California, United States of America; University of Cape Town, South Africa

## Abstract

**Background:**

Prostatitis and sexually transmitted diseases (STDs) have been positively associated with prostate cancer in previous case-control studies. However, results from recent prospective studies have been inconclusive.

**Methodogy/Principal Findings:**

We investigated the association between prostatitis, STDs, and prostate cancer among African American, Asian American, Latino, and White participants of the California Men's Health Study. Our analysis included 68,675 men, who completed a detailed baseline questionnaire in 2002–2003. We identified 1,658 incident prostate cancer cases during the follow-up period to June 30, 2006. Cox proportional hazards models were used to estimate relative risks and 95% confidence intervals. Overall, men having a history of prostatitis had an increased risk of prostate cancer than men with no history (RR = 1.30; 95% CI: 1.10–1.54). Longer duration of prostatitis symptoms was also associated with an increased risk of prostate cancer (*P* trend = 0.003). In addition, among men screened for prostate cancer (1 or 2 PSA tests), a non-significant positive association was observed between prostatitis and prostate cancer (RR = 1.10; 95% CI: 0.75–1.63). STDs were not associated with overall prostate cancer risk. In racial/ethnic stratified analysis, Latinos reporting any STDs had an increased risk of disease than those with no STDs (RR = 1.43; 95% CI: 1.07–1.91). Interestingly, foreign-born Latinos displayed a larger risk associated with STDs (RR = 1.87; 95% CI: 1.16–3.02) than U.S. born Latinos (RR = 1.15; 95% CI: 0.76–3.02).

**Conclusion:**

In summary, results from this prospective study suggest that prostatitis and STDs may be involved in prostate cancer susceptibility. While we cannot rule out the possible influence of incidental detection, future studies are warranted to further investigate the role of infectious agents related to prostatitis and STDs in prostate cancer development.

## Introduction

Prostate cancer is the most commonly diagnosed cancer among men in the United States [1]. The etiological factors driving the development of prostate cancer have yet to be fully identified and emerging evidence has suggested that infectious agents may be important risk factors for disease. Prostatitis, a commonly occurring condition that includes acute or chronic bacterial infection within the prostate [2], [3], [4], [5], and sexually transmitted diseases (STDs) [6], [7], [8], [9], [10], [11], [12] such as syphilis and gonorrhea have been linked to an increased risk of prostate cancer. It is theorized that chronic inflammation within the prostate due to the exposure of microbial agents stimulates the production of inflammatory cytokines and reactive oxygen species, leading to increased cellular proliferation and possibly carcinogenesis [13], [14].

Prior epidemiological studies have examined the role of prostatitis, STDs, and prostate cancer using primarily a case-control approach with serological studies of STDs and prostate cancer using a nested case-control design. A large meta-analysis of 29 case-control studies reported men having any history of STDs had a significant 1.5-fold increased risk of prostate cancer [6]. These collective findings suggest that STDs may be involved in prostate cancer. However, the potential for recall bias in case-control studies of gonorrhea and syphilis highlights the need for cohort studies, drawing on their prospective design to eliminate this potential bias. To date, there have been three prospective studies of self-reported prostatitis and/or STDs and prostate cancer [3], [12], [15]. The Prostate, Lung, Colorectal, and Ovarian (PLCO) Cancer Screening trial reported no association for syphilis and gonorrhea yet a borderline positive association was observed with any STDs including those identified by serological evidence [12]. The Health Professional Follow-up Study reported no overall association between prostatitis, gonorrhea, syphilis, and prostate cancer [3]. In a small nested case-control study within another Kaiser Permanente cohort study, no association was observed between prostatitis and prostate cancer, and a non-significant positive association was seen between gonorrhea and prostate cancer [15].

To further investigate the relationship between prostatitis, STDs, and prostate cancer risk, we conducted a large prospective cohort study of African American, Asian American, Latino, and White men participating in the California Men's Health Study. The sociodemographic diversity of this study population and its wide range in histories of prostatitis and STDs makes this cohort an excellent resource to examine the influence of these conditions on the development of prostate cancer.

## Methods

### Study Population

The California Men's Health Study (CMHS) is a large cohort study of 84,170 men from Northern and Southern California Kaiser Permanente. A description of the methods used to create the cohort and the characteristics of the cohort members have been published elsewhere [16]. In brief, participants between the ages of 45 and 69 years were recruited from 2002–2003 and completed a self-administered questionnaire that included information regarding demographic characteristics, health status, residential history in the United States, and lifestyle behaviors. The cohort is comprised of predominantly four racial/ethnic groups: African Americans, Asian Americans, Latinos, and Whites. This study was approved by the Institutional Review Boards of Kaiser Permanente Northern and Southern California and all study participants signed an informed consent before participating.

For the present study, we excluded men: diagnosed with any cancer prior to completion of questionnaire with the exception of localized, non-melanoma skin cancer (n = 5,608); self-reported prostate cancer (n = 8,739); had a prostatectomy prior to questionnaire (n = 16); missing prostate cancer diagnosis date (n = 1); missing race/ethnicity (n = 664); missing death date (n = 12); no active membership on the date of questionnaire (n = 995). The remaining 68,675 men were included for analysis.

### Surveillance

Cohort subjects were followed from the date of completed questionnaire until the earliest occurrence of any of the following events: prostate cancer diagnosis, radical prostatectomy, >90 day gap in Kaiser Permanente membership, death, or end of study period (June 30, 2006). Prostate cancer diagnoses were identified through Northern and Southern California Kaiser Permanente Cancer Registries, which provide data to the Surveillance, Epidemiology, and End Results (SEER) program and are considered >99% complete. Stage of disease and tumor grade were defined according to the SEER classification system. Non-aggressive and aggressive disease were defined based on stage and grade information. Localized tumors confined within the prostate that were well or moderately differentiated (SEER grade I or II) were classified as non-aggressive disease. Regional or metastatic tumors or localized tumors that were poorly differentiated or undifferentiated (SEER grade III or IV) were categorized as aggressive disease.

### Assessment of Prostatitis and Sexually Transmitted Diseases from Baseline Questionnaire

The baseline questionnaire asked subjects to report: a) have you ever had prostatitis or prostatic infection: yes, no; b) how long did the symptoms persist: <1 year, 1–2 years, 3–5 years, 6–10 years, >10 years. In addition, information on five STDs and bladder infection were assessed in response to the question “how often have you ever had any of the following diseases”: gonorrhea or ‘the clap’; syphilis; Chlamydia; genital warts (HPV); genital herpes; bladder infection. We categorized history of prostatitis as no, yes, missing and duration of prostatitis symptoms as none, <1 year, >1 year, missing. STD history was categorized for any STDs and each specific STD (no, yes, missing) and number of STDs (0, 1, >2 (multiple different STDs or repeated episodes of the same STD), missing). We included missing terms for prostatitis and STDs in all analysis; these terms demonstrated no association with risk.

### Statistical Analysis

Hazard ratios for prostate cancer incidence, referred to in this report as relative risks (RR) and 95% confidence intervals (CI) were estimated using Cox proportional hazards regression with age as the time scale. Subjects were considered to have entered the study at date of questionnaire and were followed until the earliest occurrence of any of these events: prostate cancer diagnosis, radical prostatectomy, a >90 day gap in Kaiser Permanente membership, death, or the end of the study period (June 30, 2006). All Cox models were adjusted for the following covariates that were associated with our model of prostate cancer risk: age, racial/ethnic group, family history of prostate cancer (no, yes), body mass index (BMI; <25 kg/m^2^, 25–30 kg/m^2^, >30 kg/m^2^), and income (<$20,000, $20,000–$39,999, $40,000–$59,999, $60,000–$79,999, $80,000–$99,999, >$100,000). Other potential confounders were considered such as education, smoking, ejaculation history from ages 20–29/30–39/40–49/past year, sexual orientation, diabetes, sildenafil (Viagra) use, and testosterone use. Because adjustment for these potential confounders did not notably alter risk estimates, they were not included in our final multivariable models. Tests of heterogeneity for prostatitis/STDs were conducted for severity of disease and race/ethnicity, respectively. Stratified analysis by severity of disease and race/ethnicity are presented to evaluate the consistency of effects across groups.

Stratified analyses were also conducted to evaluate effects among certain subgroups. To investigate possible detection bias due to prostate cancer screening by PSA testing, we compared associations between prostatitis, STDs, and prostate cancer among men having no PSA tests, 1 or 2 PSA tests, and 3 or more PSA tests, from the date of questionnaire to the end of follow-up based on laboratory records of PSA testing. In addition, we evaluated the effects of prostatitis and prostate cancer stratified by history BPH, a condition related to prostatitis and the incidental detection of prostate cancer. To further examine prostate cancer associations among Latinos and Asian Americans, major immigrant groups to the U.S., we compared effects between U.S. born and foreign-born men.

All statistical significance levels (*P* values) presented are two-sided. Analyses were performed using SAS 9.1 (SAS Institute, Inc., Cary, North Carolina).

## Results

Baseline characteristics of men in the study population by history of prostatitis and STDs are presented in Table 1. Men having a history of prostatitis were more likely to be White and tended to have a family history of prostate cancer, a graduate degree, higher income (>$100,000), 3 or more PSA tests, and benign prostatic hyperplasia (BPH) than those without prostatitis. Men reporting a history of any STD were more commonly African American or Latino, had a family history of prostate cancer, were current/former smokers than those without a STD history. For men with gonorrhea and syphilis, a profile generally similar to that of men with any STDs was observed with the exception that those with gonorrhea or syphilis were more likely to have only completed a high school education.

**Table 1:** Baseline charactersitics of men according to prostatitis and STD history, California Men's Health Study, 2002–2006.

		Prostatitis*		STDs*		Gonorrhea*		Syphilis*	
		No (n = 63,613)	Yes (n = 4,228)	No (n = 40,720)	Yes (n = 26,010)	No (n = 55,347)	Yes (n = 11,378)	No (n = 63,499)	Yes (n = 1,437)
Age mean (SD)		57.2 (6.9)	58.4 (6.7)	57.3 (7.0)	57.3 (6.7)	57.3 (7.0)	57.5 (6.6)	57.3 (6.9)	56.5 (6.7)
Race/ethnicity %	African American	8.4	7.5	4.5	14.5	4.8	25.8	7.6	21.8
	Asian American	9.0	4.6	11.2	4.5	9.7	3.9	8.9	6.2
	Latino	16.4	14.2	15.9	16.4	15.5	18.3	15.4	22.8
	White	61.3	71.4	62.9	61.3	65.1	48.6	63.5	45.0
	Other	4.9	2.3	5.5	3.3	4.9	3.4	4.7	4.3
Family history of prostate cancer* %	Yes	11.9	14.8	11.5	13.0	12.0	12.5	31.0	31.7
BMI %	<25 kg/m^2^	27.4	29.5	28.1	26.5	27.7	26.1	27.3	31.6
	25–30 kg/m^2^	46.0	47.8	46.0	46.4	46.0	47.0	46.3	42.4
	>30 kg/m^2^	26.6	22.7	25.9	27.1	26.3	26.9	26.4	26.0
Smoking %	never	43.6	44.3	46.8	38.5	46.2	31.5	44.2	35.9
	current	11.1	9.0	9.9	12.6	9.9	15.8	10.6	18.4
	former	45.3	46.7	43.3	49.0	43.9	52.7	45.3	45.8
Education %	< = High school	19.0	13.4	18.8	17.7	17.7	21.3	17.5	22.5
	Some college	21.1	20.6	21.7	20.2	21.7	18.5	21.7	18.3
	Bachelor's degree	35.0	31.0	33.6	36.9	33.7	40.0	33.8	35.6
	Graduate degree	25.0	35.0	26.0	25.1	26.9	20.2	27.0	23.5
Income %	<$20,000	4.8	4.3	4.4	4.8	4.2	6.1	4.3	9.0
	$20,000–$39,999	15.5	14.1	14.8	16.0	14.5	18.6	14.7	23.2
	$40,000–$59,999	19.4	17.9	19.3	19.5	19.0	21.1	19.2	22.8
	$60,000–$79,999	18.9	17.0	19.1	18.5	18.9	18.6	19.0	15.2
	$80,000–$99,999	14.0	14.2	14.2	13.9	14.3	13.2	14.3	11.8
	>$100,000	27.5	32.5	28.3	27.4	29.1	22.3	28.6	18.1
Kaiser California Region %	Northern	53.7	52.3	54.3	52.6	54.3	50.7	54.0	47.1
	Southern	46.3	47.7	45.7	47.4	45.7	49.3	46.0	52.9
Previous history of PSA testing† %	None	57.3	55.8	56.8	58.3	56.7	59.4	56.9	62.5
	1 or 2 test	27.0	23.2	27.0	26.2	27.2	24.7	27.0	21.7
	3 or more tests	15.7	21.0	16.3	15.6	16.1	16.0	16.1	15.8
History of BPH %	Yes	16.4	52.8	16.0	23.3	18.6	19.8	18.7	18.3

* Numbers do not add to 68,675 subjects due to missing responses.

† History of at least number of PSA tests from baseline to follow-up period.

Of 68,675 men in this study, 6.2% reported a history of prostatitis with 4.7% having prostatitis symptoms for less than one year (Table 2). Whites (7.2%) were more likely to report having a history of prostatitis than other racial/ethnic groups. Latinos displayed the longest duration (>1 year) of prostatitis symptoms. Overall, 26.3% of men in this study reported a history of any STD with 14.7% of men having two or more previous STDs (Table 2). The most commonly reported STD was gonorrhea (17.1%) followed by genital herpes (6.2%), genital warts (5.7%), Chlamydia (3.6%), and syphilis (2.2%). African Americans were more likely to report a history of any STD (59.9%) than Latinos (28.8%), Whites (23.7%), and Asian Americans (12.5%). Similarly, African Americans were more likely to report having multiple STDs (39.5%) with a proportion that was more than two-fold higher than that reported for any other racial/ethnic group. Gonorrhea was the most often reported STD among African Americans (52.4%) and Latinos (19.5%) while bladder infection was the most frequently reported genitourinary infection among Whites (20.5%) and Asian Americans (9.8%).

**Table 2:** Distribution of prostatitis and STDs among all men and by racial/ethnic group, California Men's Health Study, 2002–2006.

		All (N = 68,675)	African Americans (n = 5,784)	Asian Americans (n = 6,024)	Latinos (n = 11,213)	Whites (n = 42,409)	Others (n = 3,245)
Prostatitis %*	No	93.8	94.5	96.7	94.6	92.8	96.9
	Yes	6.2	5.5	3.3	5.5	7.2	3.1
Duration of prostatitis %*	None	94.0	94.9	96.9	94.8	93.0	97.0
	<1 year	4.7	3.9	2.2	3.6	5.6	2.1
	≥1 year	1.3	1.3	0.9	1.6	1.4	0.9
STDs%*	No	73.7	40.1	87.5	71.2	76.3	82.5
	Yes	26.3	59.9	12.5	28.8	23.7	17.5
Number of STDs %*	0	73.9	41.2	85.7	71.7	76.5	81.6
	1	11.4	19.3	6.0	12.4	11.1	8.1
	≥2	14.7	39.5	8.3	15.9	12.4	10.2
STDs and bladder infection*	No	61.0	32.9	79.5	60.2	61.7	72.1
	Yes	39.0	67.1	20.5	39.8	38.4	27.9
Gonorrhea %*	No	83.0	47.6	92.4	80.5	86.7	87.6
	Yes	17.1	52.4	7.6	19.5	13.3	12.4
Syphilis %*	No	97.8	93.9	98.5	96.8	98.4	98.0
	Yes	2.2	6.1	1.6	3.3	1.6	2.0
Chlamydia %*	No	96.4	90.7	98.9	97.3	96.4	98.4
	Yes	3.6	9.3	1.1	2.7	3.6	1.6
Genital warts %*	No	94.3	93.1	97.7	94.3	93.7	97.2
	Yes	5.7	6.9	2.3	5.7	6.3	2.8
Genital herpes %*	No	93.8	91.3	96.9	93.9	93.5	97.5
	Yes	6.2	8.7	3.1	6.1	6.5	2.5
Bladder infection %*	No	81.1	78.6	90.2	82.4	79.5	86.0
	Yes	18.9	21.4	9.8	17.6	20.5	14.0

* numbers do not add to 68,675 due to missing responses.

Of 68,675 cohort participants, 1,658 men were diagnosed with prostate cancer during the follow-up period from questionnaire completion to June 30, 2006. The maximum follow-up time was 4 years with a median of 2 years. Non-aggressive disease accounted for 64.8% of the cases with the remaining 35.2% of these cases having aggressive disease. Of the 1658 cases, 222 were categorized as regional/distant (SEER stages II–IV) and 450 were categorized as poorly differentiated/undifferentiated (SEER grade III/IV).

The adjusted relative risks of prostate cancer by prostatitis and STD histories are shown in Table 3. Men reporting a history of prostatitis had a significant 1.3-fold increased risk of prostate cancer in comparison to those with no history of prostatitis (95% CI: 1.10–1.54). In addition, men reporting a longer duration of prostatitis symptoms had a further increased risk of prostate cancer (*P* trend = 0.003). A significant difference was seen between the association of prostatitis and severity of disease (*P* heterogeneity = 0.03; Table 3). History of prostatitis was significantly associated with an increased risk of non-aggressive disease, yet no association was observed between prostatitis and aggressive disease. In addition, the association between prostatitis and non-aggressive prostate cancer remained significant with adjustment of number of PSA tests (RR = 1.52; 95% CI: 1.25–1.86). There was no overall association between history of any STDs and prostate cancer. Of the five specific STDs examined, a history of genital warts was inversely associated with prostate cancer risk (RR = 0.77; 95% CI: 0.60–0.99).

**Table 3:** Multivariate relative risks and 95% CIs of prostate cancer associated with prostatitis and STD history by severity of disease, California Men's Health Study, 2002–2006*.

		Total Pca†		non Aggressive Pca‡		Aggressive PCa^§^	
		No. of Cases	RR (95% CI)	No. of Cases	RR (95% CI)	No. of Cases	RR (95% CI)
Prostatitis	No	1484	1.00	924	1.00	524	1.00
	Yes	147	1.30 (1.10–1.54)^a^	108	1.52 (1.25–1.86)^b^	39	1.00 (0.72–1.39)
Duration of prostatitis n (%)	None	1484	1.00	924	1.00	524	1.00
	<1 year	103	1.22 (1.00–1.49)^c^	79	1.49 (1.18–1.88)^e^	24	0.82 (0.54–1.24)
	≥1 year	36	1.50 (1.08–2.08)^d^	29	1.47 (0.96–2.24)	14	1.67 (0.98–2.84)
STDs	No	1142	1.00	730	1.00	391	1.00
	Yes	464	1.02 (0.91–1.15)	288	1.00 (0.86–1.15)	163	1.06 (0.87–1.29)
Number of STDs n (%)	0	1142	1.00	730	1.00	391	1.00
	1	195	1.06 (0.90–1.23)	122	1.03 (0.85–1.26)	67	1.08 (0.83–1.34)
	≥2	232	1.00 (0.87–1.15)	145	1.00 (0.83–1.21)	83	1.08 (0.84–1.39)
STDs & bladder infection	No	920	1.00	572	1.00	332	1.00
	Yes	686	1.04 (0.94–1.15)	445	1.09 (0.96–1.24)	222	0.94 (0.79–1.12)
Gonorrhea	No	1274	1.00	812	1.00	437	1.00
	Yes	345	1.07 (0.94–1.21)	216	1.06 (0.90–1.25)	118	1.05 (0.84–1.31)
Syphilis	No	1516	1.00	961	1.00	524	1.00
	Yes	43	1.23 (0.90–1.67)	28	1.28 (0.87–1.88)	15	1.25 (0.74–2.10)
Chlamydia	No	1485	1.00	947	1.00	507	1.00
	Yes	54	1.05 (0.80–1.39)	31	0.93 (0.64–1.34)	23	1.37 (0.90–2.10)
Genital warts	No	1479	1.00	936	1.00	511	1.00
	Yes	63	0.77 (0.60–0.99)^f^	40	0.77 (0.56–1.06)	23	0.83 (0.54–1.26)
Genital herpes	No	1465	1.00	928	1.00	507	1.00
	Yes	78	0.86 (0.69–1.08)	46	0.88 (0.59–1.08)	30	0.99 (0.68–1.43)
Bladder infection	No	1233	1.00	759	1.00	449	1.00
	Yes	320	1.02 (0.90–1.15)	220	1.13 (0.97–1.32)	92	0.82 (0.65–1.02)

* Adjusted for age, race, family history, BMI, and income.

† Numbers do not add add to 1,658 cases due to missing responses.

‡ Numbers do not add add to 1,051 cases due to missing responses.

‡ Numbers do not add add to 871 cases due to missing responses.

a *P* = 0.003.

b *P* = <.0001.

c *P* = .051.

d *P* = 0.017.

e *P* = 0.007.

f *P* = 0.044.

In racial/ethnic-specific analysis, Latinos and Whites had a significantly increased risk of prostate cancer associated with both prostatitis history and duration (Table 4). Latinos and Whites had a 1.8 to 2-fold significantly increased risk of prostate cancer among those having prostatitis symptoms for >1 year in comparison to those with no symptoms (Latino *P* trend = 0.044; Whites *P* trend = 0.002). Latinos also had a significantly increased risk of prostate cancer associated with history of any STDs (RR = 1.43; 95% CI: 1.07–1.91) and gonorrhea (RR = 1.39; 95% CI: 1.01–1.91). Asian Americans experienced a significant positive association with history of syphilis and Chlamydia, while the Other racial/ethnic group had a significantly increased risk associated with bladder infections. Notably, among African Americans, where 5.5% reported a history of prostatitis and 59.9% reported a history of STDs, there were no associations for either prostatitis or STDs and risk of prostate cancer.

**Table 4:** Multivariate relative risks and 95% CIs of prostate cancer associated with prostatitis and STD history by racial/ethnic group, California Men's Health Study, 2002–2006.

		African Americans		Asian Americans		Latinos		Whites		Others		*P* het†
		No. of Cases	RR (95% CI)	No. of Cases	RR (95% CI)	No. of Cases	RR (95% CI)	No. of Cases	RR (95% CI)	No. of Cases	RR (95% CI)	
Prostatitis	No	253	1.00	82	1.00	191	1.00	902	1.00	56	1.00	0.270
	Yes	14	0.79 (0.46–1.36)	7	1.95 (0.89–4.24)	20	1.62 (1.02–2.57)^a^	101	1.31 (1.07–1.62)^b^	5	1.85 (0.66–5.20)	
Duration of prostatitis	none	253	1.00	82	1.00	191	1.00	902	1.00	56	1.00	0.230
	<1 year	9	0.77 (0.39–1.50)	5	2.11 (0.85–5.26)	10	1.27 (0.67–2.41)	74	1.23 (0.97–1.56)	5	2.49 (0.88–7.05)	
	≥1 year	2	0.42 (0.11–1.71)	2	2.01 (0.49–8.24)	8	2.05 (1.01–4.17)^c^	24	1.75 (1.16–2.62)^d^	1	–	
STDs	No	100	1.00	76	1.00	135	1.00	780	1.00	51	1.00	0.2711
	Yes	164	1.04 (0.81–1.36)	13	1.26 (0.70–2.28)	72	1.43 (1.07–1.91)^e^	205	0.92 (0.79–1.07)	10	1.04 (0.52–2.06)	
Number of STDs	0	100	1.00	76	1.00	135	1.00	780	1.00	51	1.00	0.505
	1	50	1.04 (0.74–1.46)	6	1.24 (0.54–2.85)	35	1.60 (1.10–2.33)^f^	99	0.94 (0.76–1.16)	5	1.23 (0.49–3.09)	
	≥2	101	1.05 (0.80–1.39)	7	1.60 (0.73–3.50)	29	1.43 (0.96–2.15)	93	0.91 (0.74–1.13)	2	0.52 (0.13–2.17)	
STDs and bladder infection	No	80	1.00	73	1.00	109	1.00	620	1.00	38	1.00	0.092
	Yes	182	1.05 (0.81–1.37)	17	0.89 (0.52–1.51)	100	1.39 (1.06–1.82)^g^	365	0.97 (0.85–1.10)	22	1.58 (0.93–2.70)	
Gonorrhea	No	111	1.00	81	1.00	156	1.00	875	1.00	51	1.00	0.342
	Yes	153	1.12 (0.88–1.44)	8	1.16 (0.56–2.41)	51	1.39 (1.01–1.91)^h^	124	0.94 (0.78–1.14)	9	1.43 (0.70–2.92)	
Syphilis	No	223	1.00	84	1.00	182	1.00	969	1.00	58	1.00	0.419
	Yes	17	1.32 (0.80–2.17)	4	3.72 (1.35–10.26)^i^	8	1.38 (0.64–2.93)	13	0.93 (0.54–1.61)	1	0.91 (0.12–6.66)	
Chlamydia	No	216	1.00	83	1.00	181	1.00	949	1.00	56	1.00	0.148
	Yes	18	1.00 (0.62–1.63)	3	5.55 (1.70–18.09)^j^	6	1.82 (0.80–4.15)	26	0.88 (0.59–1.32)	1	1.39 (0.19–10.18)	
Gential warts	No	225	1.00	87	1.00	183	1.00	927	1.00	57	1.00	0.696
	Yes	9	0.55 (0.28–1.07)	0	–	4	0.44 (0.16–1.19)	49	0.93 (0.70–1.25)	1	0.58 (0.08–4.25)	
Genital herpes	No	218	1.00	85	1.00	175	1.00	931	1.00	56	1.00	0.946
	Yes	19	0.91 (0.57–1.46)	3	1.20 (0.38–3.81)	11	1.16 (0.63–2.13)	44	0.78 (0.58–1.06)	1	0.75 (0.10–5.54)	
Bladder infection												
	No	191	1.00	82	1.00	151	1.00	766	1.00	43	1.00	0.413
	Yes	47	0.90 (0.65–1.24)	7	0.73 (0.34–1.58)	38	1.06 (0.74–1.52)	214	1.03 (0.88–1.20)	14	1.96 (1.06–3.61)^k^	

* adjusted for age, family history, BMI, and income.

† *P* heterogenity by racial/ethnic group.

a *P* = 0.043.

b *P* = 0.010.

c *P* = 0.048.

d *P* = 0.007.

e *P* = 0.016.

f *P* = 0.015.

g *P* = 0.025.

h *P* = 0.043.

i *P* = 0.009.

j *P* = 0.005.

k *P* = 0.031.

Since many Latinos and Asian Americans are immigrants to the U.S., we explored whether their risks associations differed by place of birth (Table 5). For Latinos, we observed larger relative risks for history of prostatitis and STDs among those foreign-born. In particular, a significant 1.9-fold increased risk of prostate cancer was associated with history of STDs among Latinos born in either Mexico or Central/South America (*P* = 0.014) while no association was seen among Latinos born in the United States. Gonorrhea, a more symptomatic STD, was significantly associated with prostate cancer risk among foreign-born Latinos (RR = 1.95; 95% CI: 1.20–3.16) but not among U.S. born Latinos. For Asian Americans, we observed a similar pattern as seen with Latinos such that larger effects were detected among those foreign-born in comparison to those born in the United States. A significant association between prostatitis and prostate cancer was seen for foreign-born Asian Americans (RR = 2.66; 95% CI: 1.02–6.95).

**Table 5:** Latino relative risks and 95% CIs of prostate cancer associated with prostatitis and STD history by place of birth, California Men's Health Study, 2002–2006.

		Latinos				Asians			
		Born in U.S. (n = 6,042)		Foreign-born† (n = 4,611)		Born in U.S. (n = 2,264)		Foreign-born† (n = 3,245)	
		No. of Cases	RR (95% CI)	No. of Cases	RR (95% CI)	No. of Cases	RR (95% CI)	No. of Cases	RR (95% CI)
Prostatitis	No	118	1.00	65	1.00	44	1.00	33	1.00
	Yes	10	1.39 (0.73–2.67)	8	1.62 (0.77–3.41)	0	–	5	2.66 (1.02–6.95)^a^
STDs	No	93	1.00	34	1.00	40	1.00	33	1.00
	Yes	33	1.15 (0.76–1.72)	35	1.87 (1.16–3.02)^b^	4	0.66 (0.24–1.86)	5	1.24 (0.48–3.21)
Number of STDs	0	93	1.00	34	1.00	40	1.00	33	1.00
	1	17	1.16 (0.68–1.98)	17	2.46 (1.36–4.44)^c^	2	0.58 (0.14–2.42)	3	1.94 (0.59–6.38)
	≥2	13	1.25 (0.70–2.25)	14	1.75 (0.93–3.28)	2	0.83 (0.20–3.51)	2	1.06 (0.25–4.50)
Gonorrhea	No	107	1.00	40	1.00	42	1.00	36	1.00
	Yes	19	1.02 (0.63–1.67)	29	1.95 (1.20–3.16)^d^	2	0.47 (0.11–1.97)	2	0.71 (0.17–2.98)

* adjusted for age, family history, BMI, and income.

† Born in Mexico, Central/South America.

a P = 0.045.

b *P* = 0.014.

c *P* = 0.003.

d *P* = 0.007.

† Born in Asia.

In a stratified analysis of PSA screening to examine the potential influence of detection bias, prostatitis displayed positive associations with prostate cancer risk across all strata of number of previous laboratory PSA tests during follow-up with significant associations seen for men having no prior PSA testing and those having 3 or more PSA tests, RR = 1.34; 95% CI: 1.05–1.70 and RR = 1.42; 95% CI: 1.03–1.94, respectively (*P* heterogeneity = 0.215; Table 6). Adjusting both for the time-fixed number of previous PSA tests during follow-up as well as time-dependent number of PSA tests during follow-up, prostatitis remained significantly associated with prostate cancer risk (RR = 1.30; 95% CI: 1.10–1.55 and RR = 1.25; 95% CI: 1.03–1.52, respectively). In addition, we found no evidence of heterogeneity by age (<55 years and >55 years) among men having 1 or 2 PSA tests, more likely representing those who are routinely screened (*P* heterogeneity = 0.607). Prostatitis was not associated with prostate cancer among men with no history of BPH (RR = 1.07; 95% CI: 0.78–1.47) as well as among men with a history of BPH (RR = 1.16; 95% CI: 0.93–1.44). For STDs, we observed no difference by PSA testing (data not shown). Among foreign-born Latinos, STD history was significantly associated with prostate cancer among those with no prior PSA screening (RR = 2.16; 95% CI: 1.20–3.87) and non-significant positive associations were observed among men having 1 or 2 PSA tests and 3 or more tests, RR = 1.33; 95% CI: 0.46–3.86 and RR = 3.51; 95% CI: 0.58–21.02, respectively, with a *P* heterogeneity = 0.491.

**Table 6:** Multivariate relative risks and 95% CIs of prostate cancer associated with prostatitis and STD history by number of PSA tests during follow-up, California Men's Health Study, 2002–2006*.

		No. of PSA tests during follow-up						
		None		1 or 2		3 or more		*P* het
		No. of Cases	RR (95% CI)	No. of Cases	RR (95% CI)	No. of Cases	RR (95% CI)	
Prostatitis	No	751	1.00	400	1.00	333	1.00	0.215
	Yes	75	1.34 (1.05–1.70)^a^	27	1.10 (0.75–1.63)	45	1.42 (1.03–1.94)^b^	
Duration of prostatitis n (%)	None	751	1.00	400	1.00	333	1.00	
	<1 year	48	1.15 (0.85–1.54)	24	1.36 (0.90–2.06)	31	1.28 (0.88–1.85)	0.071
	≥1 year	20	1.71 (1.09–2.66)^c^	3	0.52 (0.17–1.61)	13	2.08 (1.19–3.63)^d^	

* Adjusted for age, race, family history, BMI, and income.

a *P* = 0.018.

b *P* = 0.029.

c *P* = 0.019.

d *P* = 0.010.

## Discussion

In the current study, we investigated whether prostatitis and STDs influenced the risk of prostate cancer among men enrolled in the California Men's Health Study. In this multiethnic study of over 68,000 men, overall a history of prostatitis and longer duration of prostatitis symptoms was associated with an increased risk of prostate cancer. Overall, STD history was not associated with prostate cancer. In racial/ethnic stratified analysis, STD history was associated with prostate cancer risk among Latinos with those foreign-born experiencing a greater risk of prostate cancer than those born in the United States.

Prostatitis includes several conditions that are categorized by the National Institutes of Health as: (I) acute bacterial prostatitis; (II) chronic bacterial prostatitis; (III) chronic prostatitis/chronic pelvic pain syndrome (a) inflammatory and (b) non-inflammatory; (IV) asymptomatic inflammatory prostatitis [17]. These conditions present with various genitourinary symptoms, causing men to seek medical attention that may result in the incidental detection of prostate cancer through PSA testing and digital rectal exams. A key concern is whether detection bias could have influenced the association we observed between history of prostatitis and prostate cancer risk. To address this issue, we conducted stratified analysis by laboratory records of number of PSA tests during follow-up as well as by history of BPH, which is also associated with incidental detection of prostate cancer. Our finding of no evidence of heterogeneity in effects between prostatitis and prostate cancer across strata of no PSA test (note, 80% of the 826 cases within this strata had a record of PSA testing within a five year period prior to baseline), 1–2 PSA test(s), and 3 or more PSA tests would suggest that our overall positive association may not be significantly influenced by detection issues. However, there are limitations in this analysis given the small numbers per stratum that may result in chance findings and/or limited ability to detect effects such as seen for the stratum of 1–2 PSA tests (n = 27 prostate cases reporting history of prostatitis). In particular, the stratum of 1–2 PSA tests would likely represent men undergoing routine screening with an equal opportunity for prostate cancer detection—with the lack of a clear association among this subgroup, results concerning our effort to evaluate detection bias should be interpreted with caution. These issues also apply to our evaluation of duration of prostatitis symptoms in which the positive association we observed may be biased due to increased opportunity of prostate cancer detection with greater length of contact with physicians. Again, in our stratified analysis by number of PSA tests, there is the suggestion that there may be no differences across the levels of PSA testing; yet, we cannot completely rule out such bias. In addition, the lack of an association between prostatitis and prostate cancer in our stratified analysis by BPH further raises the possibility that detection bias may be operating in our study. BPH typically leads to higher levels of PSA, which in turn may increase a man's likelihood of having a prostate biopsy and subsequent detection of prostate cancer. The attenuation of association between prostatitis and prostate cancer within strata of BPH could suggest that our overall positive association between prostatitis and prostate cancer may be largely influenced by the presence of BPH and its affiliated increased likelihood of prostate cancer detection. Furthermore, the significant association seen between prostatitis and non-aggressive disease, yet no association between prostatitis and aggressive disease also highlight possible biases due to detection issues. We would expect the effect of prostatitis to behave similarly for both strata of disease severity. It is possible the association between prostatitis and non-aggressive disease, low grade and localized tumor types often identified by prostate cancer screening, may actually reflect a higher likelihood of identifying screen detected disease among those with a history of prostatitis. While our findings between prostatitis and prostate cancer may represent a false positive association, our findings do warrant additional research to further evaluate the relationship between prostatitis and prostate cancer with careful attention to underlying influences of possible detection bias.

The myriad of conditions of prostatitis differ in prevalence as well as their associations with infectious etiology. Chronic prostatitis/chronic pelvic pain syndrome (category III) is the most common (>90%) and includes both inflammatory (30%–45%) and non-inflammatory (45%–60%) forms, while acute and chronic bacterial prostatitis (categories I and II) and asymptomatic inflammatory prostatitis (category IV) are less common (∼10%) and are associated with inflammation [17], [18], [19]. With a substantial proportion of non-inflammatory chronic prostatitis having an unclear etiology and no association with inflammatory pathways, the contribution of prostatitis to prostate cancer development may not necessarily act through a chronic inflammation model. To better clarify the underlying etiologic processes, future work should evaluate the specific four categories of prostatitis in relationship to prostate cancer risk.

The prevalence of prostatitis in our study (6.2%) is similar to that estimated by a recent meta-analysis of five population-based studies (8.2%) [20] and a previous study of Kaiser Permanente Northwest (4.5%) [21]. However, our prevalence among Whites (7.2%) is much lower than that observed among men in the HPFS (15.9%) [3], the majority of whom are White health professionals. This difference between cohorts is likely due to the health professionals in the HPFS having greater knowledge and awareness of prostatitis conditions and a lesser degree of misclassification of prostatitis history than our more general population of California men. The Health Professionals Follow-up Study (HPFS) [3] observed no overall association between prostatitis and prostate cancer yet a significant increased risk of disease among younger men reporting being routinely screened (<55 years; RR = 1.49; 95%: 1.08–2.06; *P* interaction = 0.006). In contrast, our study observed a significant positive overall association between prostatitis and prostate cancer and no evidence of interaction by age among screened men having 1 or 2 PSA tests (*P* interaction = 0.607). Possible explanations for these differences across studies may be due to differences in the prevalence and assessment of PSA screening. First, the HPFS has a high prevalence of PSA self-reported screening (∼90%) in contrast to the CMHS laboratory testing of PSA (∼43%). The lower prevalence of PSA testing in the CMHS limits our ability to detect effects among those screened as well as raises issues related to the influence of detection bias as discussed previously. Second, the HPFS distinguished between routine screening of prostate cancer versus testing due to symptomatic conditions based on self-report data. In contrast, we are unable to make such a distinction in our use of laboratory records of PSA tests. Although in our stratification of no PSA tests, 1–2 PSA test(s), and 3 or more PSA tests, we expect the stratum of 1–2 PSA test(s) to be most similar to the routinely screened group from the HPFS in which a null association was observed, again we were limited here with smaller sample size, influencing our ability to detect effects.

The racial/ethnic patterns of STD history among men in our study mirror the nationwide trends in disease. The Centers for Disease Control and Prevention (CDC) report higher rates of STDs among racial/ethnic minorities in comparison to Whites, with the exception of Asian/Pacific Islanders [22]. Similarly, in our study a disproportionate number of African Americans reported having STDs in comparison to other racial/ethnic groups. In our study, we observed no overall association between any STDs and prostate cancer, and these findings are similar to reports from PLCO trial and HPFS that found no association between syphilis and gonorrhea and prostate cancer [3], [12].

Interestingly, the positive associations between STD history (and gonorrhea) and prostate cancer among Latinos suggest there may be racial/ethnic-specific effects in spite of the lack of statistical evidence of heterogeneity across groups. Moreover, foreign-born Latinos displayed elevated risks of prostate cancer associated with STD history. Whether these findings may have been biased due to detection issues related to prostate cancer screening, the positive associations seen across strata of number of PSA tests and lack of evidence of heterogeneous effects would suggest no large scale bias among this particular subgroup, although we recognize the lack of significant findings in all of the strata limits any clear conclusion. Alternatively, these findings may suggest that STDs acquired outside of the U.S. may be associated with an increased susceptibility of prostate cancer. Similar to Latinos, Asian Americans another recent immigrant group to the U.S. displayed a comparable pattern of higher risk of prostate cancer associated with STDs among those foreign-born. These findings further support the observations that STDs acquired outside of the U.S. may be related to disease. It is possible that untreated or delayed-treatment of STDs in less developed countries allow for prolonged infection of microbial agents and chronic inflammation, ultimately leading to prostate cancer development. This has been previously proposed by Sutcliffe et al. [3], who suggested that repeated episodes of gonorrhea (or co-infections) and/or those untreated or have delayed treatment may be associated with prostate cancer risk among populations having a higher burden of sexually transmitted infections. STDs such as gonorrhea, caused by *Neisseria gonorrhoeae* bacterium, have been shown to infect the prostate and elicit an inflammatory response. In the scenario, where *Neisseria gonorrhoeae* is able to establish a chronic inflammation within the prostate by either insufficient immune response, repeated infections, or delay/lack of treatment, the local production of inflammatory cytokines (e.g. interleukin-6 and interleukin-8) and reactive oxygen species as well as recruitment of neutrophils and macrophages that carry inflammatory agents (e.g. myloperoxidase and nitric oxidase) may induce cell and genome damage leading to increased cell proliferation. This inflammatory cascade in response to STD infection may ultimately lead to carcinogenesis within the prostate.

Our current study has several strengths. The prospective design minimizes differential recall between cases and controls and the extensive information from baseline questionnaires allowed for the evaluation of numerous confounding factors. Our large multiethnic study enabled us to explore whether risk associations differed across diverse racial/ethnic groups. Furthermore, this study population has a wide range in history of prostatitis and STDs that likely reflect the general population of California men. Although this is not a geographical population-based study, Kaiser Permanente provides medical care to over 6.3 million people in California, representing ∼25–30% of the general population it serves. The relative equal access to care lessens issues related to availability of diagnostic services for prostatitis, STDs, and prostate cancer.

However, there are several limitations to our study. We did not have information on history of prostatitis/STDs as a time-dependent variable and relied on baseline information as an approximation of relevant prostatitis/STDs history. This poses an interesting question for future studies to evaluate the time course of prostatitis/STDs infection on prostate cancer risk, particularly among a young cohort of men. In addition, chance cannot be ruled out, especially with smaller numbers in our stratified analysis given our relatively short follow-up time in spite of our large cohort size. Furthermore, we did not have information on the underlying etiology of prostatitis and identifying the specific subtype (i.e. bacterial, viral, chronic pelvic pain syndrome) may clarify the association reported here.

In conclusion, our study results are consistent with the hypothesis that chronic inflammation and infectious agents related to prostatitis and STDs may be involved in prostate cancer susceptibility. Presently, there are no established modifiable risk factors for prostate cancer. If future studies can identify infectious agents as etiological factors in prostate cancer development, important public health measures can be made in reducing the burden of this common disease.

## References

[1] American Cancer Society (2008). Cancer Facts and Figures..

[2] Dennis LK, Lynch CF, Torner JC (2002). Epidemiologic association between prostatitis and prostate cancer.. Urology.

[3] Sutcliffe S, Giovannucci E, De Marzo AM, Leitzmann MF, Willett WC (2006). Gonorrhea, syphilis, clinical prostatitis, and the risk of prostate cancer.. Cancer Epidemiol Biomarkers Prev.

[4] Patel DA, Bock CH, Schwartz K, Wenzlaff AS, Demers RY (2005). Sexually transmitted diseases and other urogenital conditions as risk factors for prostate cancer: a case–control study in Wayne County, Michigan.. Cancer Causes Control.

[5] Roberts RO, Bergstralh EJ, Bass SE, Lieber MM, Jacobsen SJ (2004). Prostatitis as a risk factor for prostate cancer.. Epidemiology.

[6] Taylor ML, Mainous AG, Wells BJ (2005). Prostate cancer and sexually transmitted diseases: a meta-analysis.. Fam Med.

[7] Dennis LK, Dawson DV (2002). Meta-analysis of measures of sexual activity and prostate cancer.. Epidemiology.

[8] Fernandez L, Galan Y, Jimenez R, Gutierrez A, Guerra M (2005). Sexual behaviour, history of sexually transmitted diseases, and the risk of prostate cancer: a case-control study in Cuba.. Int J Epidemiol.

[9] Hayes RB, Pottern LM, Strickler H, Rabkin C, Pope V (2000). Sexual behaviour, STDs and risks for prostate cancer.. Br J Cancer.

[10] Lightfoot N, Conlon M, Kreiger N, Sass-Kortsak A, Purdham J (2004). Medical history, sexual, and maturational factors and prostate cancer risk.. Ann Epidemiol.

[11] Rosenblatt KA, Wicklund KG, Stanford JL (2001). Sexual factors and the risk of prostate cancer.. Am J Epidemiol.

[12] Huang WY, Hayes R, Pfeiffer R, Viscidi RP, Lee FK (2008). Sexually transmissible infections and prostate cancer risk.. Cancer Epidemiol Biomarkers Prev.

[13] Coussens LM, Werb Z (2002). Inflammation and cancer.. Nature.

[14] De Marzo AM, Platz EA, Sutcliffe S, Xu J, Gronberg H (2007). Inflammation in prostate carcinogenesis.. Nat Rev Cancer.

[15] Hiatt RA, Armstrong MA, Klatsky AL, Sidney S (1994). Alcohol consumption, smoking, and other risk factors and prostate cancer in a large health plan cohort in California (United States).. Cancer Causes Control.

[16] Enger SM, Van den Eeden SK, Sternfeld B, Loo RK, Quesenberry CP (2006). California Men's Health Study (CMHS): a multiethnic cohort in a managed care setting.. BMC Public Health.

[17] Krieger JN, Nyberg L, Nickel JC (1999). NIH consensus definition and classification of prostatitis.. JAMA.

[18] Schaeffer AJ, Knauss JS, Landis JR, Propert KJ, Alexander RB (2002). Leukocyte and bacterial counts do not correlate with severity of symptoms in men with chronic prostatitis: the National Institutes of Health Chronic Prostatitis Cohort Study.. J Urol.

[19] True LD, Berger RE, Rothman I, Ross SO, Krieger JN (1999). Prostate histopathology and the chronic prostatitis/chronic pelvic pain syndrome: a prospective biopsy study.. J Urol.

[20] Krieger JN, Lee SW, Jeon J, Cheah PY, Liong ML (2008). Epidemiology of prostatitis.. Int J Antimicrob Agents.

[21] Clemens JQ, Meenan RT, O'Keeffe-Rosetti MC, Gao SY, Brown SO (2006). Prevalence of prostatitis-like symptoms in a managed care population.. J Urol.

[22] (2006). Trends in Reportable Sexually Transmitted Diseases in the United States, 2006 National Surveillance Data for Chlamydia, Gonorrhea, and Syphilis..

